# A Comprehensive Tobacco Control Policy Program in a Mining Industry in Indonesia: Did It Work?

**DOI:** 10.3389/fpubh.2022.853862

**Published:** 2022-03-24

**Authors:** Yayi S. Prabandari, Bagas S. Bintoro, Purwanta Purwanta

**Affiliations:** ^1^Department of Health Behavior, Environment, and Social Medicine, Faculty of Medicine, Public Health, and Nursing, Universitas Gadjah Mada, Yogyakarta, Indonesia; ^2^Center of Health Behavior and Promotion, Faculty of Medicine, Public Health, and Nursing, Universitas Gadjah Mada, Yogyakarta, Indonesia; ^3^Department of Mental Health and Community Health Nursing, Faculty of Medicine, Public Health, and Nursing, Universitas Gadjah Mada, Yogyakarta, Indonesia

**Keywords:** smoke-free policy, worksite, mining, Indonesia, Kaltim Prima Coal

## Abstract

**Background:**

Risk factor controls, including smoking cessation and prevention, impact health costs. This study aimed to describe the Kaltim Prima Coal (KPC), one of Indonesia's largest coal mining operations, comprehensive tobacco control policy program in 2015 and its impact on smoking behavior among the employees.

**Method:**

A survey among 404 employees was conducted to assess the impact of the smoke-free KPC programs. In addition to the descriptive analysis, logistic regression was used to measure the association of intention to the smoking behavior change and the association between intention and the determinants using the Theory of Planned Behavior in 102 smokers.

**Results:**

A series of tobacco control programs: advocacy, health education, brief interventions for smoking cessation, peer counselor training, media campaigns, and policy regulations were implemented. About 95.5% of the respondents attended the KPC Smoke-Free 2015 programs, and 97.8% reported they already knew that KPC is a total smoke-free area. Nearly 50% of the respondents expressed that the staff complied with the rules and no longer smoked in KPC. Majority of smokers (76.6%) reduced their consumption, and 5.6% of them quit smoking. Among smokers, we found that attitude toward smoking cessation, subjective norm, and perceived control for quitting were related to the intention to stop smoking.

**Conclusions:**

The KPC smoke-free policy has been comprehensively implemented. Regulations on smoking and tobacco controls should be maintained, and monitoring should be consistently done. Media campaigns on the regulations and the availability of trained peer educators for smoking cessation help need to be applied continuously.

## Introduction

### Background

Indonesia is one of the most populous smoker countries in the world. The Basic Health Research in Indonesia reported that the prevalence of smokers aged >10 in 2018 is 28.9%, and 55.8% of males are smokers (1). The number of men smokers is among the highest globally (2). The average number of cigarettes consumed in Indonesia is 12.8 cigarettes/day (the equivalent of one pack of commonly-sold cigarettes in Indonesia) (1). In 2017, there were 2,25,720 deaths caused by tobacco in Indonesia, while 1,47,510 were due to tobacco-related cardiovascular disease (CVD) (3).

Tobacco is considered the second most common risk factor for death and disability and a contributing factor for the three leading causes of death in Indonesia, which are stroke, ischemic heart disease, and diabetes (4). Smoking has been proven to be harmful to health, causing lung problems such as emphysema, CVD, heart attacks, leading to premature death (5, 6). Smoking also worsens sperm quality for men, contributing to infertility problems (7).

The discussion about smoking has been expanding to concerns about secondhand smoke (SHS) exposure since 50 years ago. The debate started in 1972, but the conclusion is still the same: exposure to SHS or Environment Tobacco Smoke (ETS) is harmful (5, 8). A significantly increased risk of severe dementia syndromes was reported among people exposed to ETS (9). People exposed to ETS at their workplace were reported 37% more likely to have visited a doctor for a respiratory illness (10). Several studies showed no risk-free level of exposure to SHS (5, 8). Almost 75% of adults aged >15 years old in Indonesia were exposed to ETS (11). About 20% of non-smoker adult workers were exposed to SHS in the workplace (12). Exposure to SHS is a cause of many illnesses. Homes and workplaces are where the most exposure to SHS occurs. Complete elimination of tobacco smoke protects non-smokers from exposure to SHS (13), while partial bans on smoking cannot eliminate exposures of non-smokers to SHS (8). Meanwhile, one study found that implementing the smoke-free law in Thailand decreased acute myocardial infarction hospitalization by 13% among adults <45 years old (14).

Existing findings from recent studies indicate that smoke-free legislations provide benefits in many ways (15–17), i.e., reducing SHS, less smoking prevalence, and cessation (18). Implementing a full restriction policy is the only effective way to ensure that SHS exposure prevention is successfully implemented in the workplace (8, 19). Economically, smoking-related costs can be reduced by establishing smoke-free workplaces (20).

Smoking cessation programs can be part of the smoke-free workplace as a complement to support employees to comply with the zero-tolerance of tobacco policy (21). A review of the systematic review of smoking cessation in the workplace indicated that several interventions combined (six trials; 5,018 participants) helped people to stop smoking (22). Smoke-free workplaces have been indicated to encourage employees to stop smoking; however, the mechanism of behavior change is not well-understood. A theoretical framework such as the theory of planned behavior (TPB) can be applied to evaluate a smoking intervention (23), as reported by Ajzen that the TPB has been widely used in health research and cited more than 4,000 times by google scholar in 2010 (24). The TPB consists of three main constructs: attitude toward behavior, subjective norm, and perceived control, which contribute to intention to perform the behavior (25). Where attitudes and subjective norms are favorable and perceived control is high, a strong intention to perform the behavior should occur. The behavior will be formed as a result of strong intention. Fong et al. (26) used the TPB as one of the primary theories underlying the impact of countries' level of tobacco control policy on smoking behavior. Moreover, Macy et al. (27) used structural equation modeling to conduct a theory of planned behavior analysis with data from 395 smokers living in seven Texas cities, three with a comprehensive smoke-free air law and four without a comprehensive law. The result showed that smoke-free air laws appear to influence quitting intentions by forming positive attitudes about regulating smoking in public places and the perception of normative pressure to take measures to quit. This paper is sought to assess the impact of a comprehensive tobacco policy program in a big mining company on smoking behavior and analyze the determinant factors related to the change of smoking behavior based on the theory of planned behavior.

### Setting and Intervention

PT Kaltim Prima Coal (KPC) is located in East Kalimantan. Starting its exploration in 1982, KPC currently employs 25,363 people (4,947 of PT KPC and 20,416 from contractors). Smoking prevalence among employees of KPC reached 49% in 2010 and only fell very slightly to 46% in 2013. Before the Governor of East Kalimantan Regulation on Smoke-Free Areas was enacted in 2010, KPC had already started implementing a smoke-free zone. The implementation was made after three employees died within 2 months due to smoking-related diseases.

In January 2014, KPC management launched a comprehensive smoke-free policy, including the quit smoking program named “Smoke-Free KPC 2015” (*KPC Bebas Rokok 2015*). KPC has applied several rules and regulations to accelerate that program, such as random proactive inspections of workers to check whether workers are still carrying cigarettes into the working site. KPC has also conducted health education for employees started from high management up to the laborers and the employees' wife association and community surrounding the mining. Outdoor media and videos were also applied as health education messages. There is a feedback system between this smoke-free program and its environment for evaluation purposes, and fidelity assessments need to be routinely conducted to determine its effectiveness (28). Twenty employees were trained as peer counselors for smoking cessation in the education and outreach programs.

## Materials and Methods

This cross-sectional study used a self-reported questionnaire as the data collection tool. The population of this study was KPC workers, and the participants were 404 workers. Knowing that KPC consists of several divisions, stratification sampling was done using data from the human resource department of KPC. Ethical approval was obtained from the Faculty of Medicine's Medical and Health Research Ethics Committee, Universitas Gadjah Mada. All of the participants completed a written informed consent form regarding the goals of the study and the willingness to participate in the study. Variables asked to the smoker participants concerned about their smoking habit and cigarette consumption change, while knowledge and attitudes on smoke-free policy variables were obtained from all participants. Questions for smoking habits were: “How often did you smoke a cigarette this month?” and “How many sticks do you smoke per day?”. Smokers' smoking behavior change was measured by the tobacco consumption change in general and during their working time inside KPC. The options of those questions were “no change,” “increase,” “decrease,” or “stop smoking.” Knowledge (awareness) of the smoke-free policy was based on questions with options of “yes” and “no.” We also asked the respondents' views on policy implementation of KPC' SFA Regulation in a five-scale Likert scale.

In addition to descriptive analysis, we developed a model using the Theory of Planned Behavior to measure the association of intention (no intention to stop and intended to stop) to the smoking behavior change (“no change and increase” and “decrease or stop smoking.”). We also measured the association between intention and the determinants, namely knowledge (total score of knowledge of the smoke-free policy, 0–3), attitude (total score of respondents' views on policy implementation of KPC' SFA Regulation, 0–8), subjective norm (total score of respondents' views on the support of family on the smoking cessation, the intention of smoking cessation because of family, and KPC policy supporting them to stop smoking; 0–3), and perceived behavioral control (total score of respondents' views on the benefit of the smoking cessation, the statement on the duration of having smoking cessation intention, self-confidence on stop smoking; 0–3). Cronbach's alpha was used to examine the reliability of all instrument constructs. The Cronbach's alpha value for the scale of knowledge (α = 0.7), attitude (α = 0.7), subjective norm (α = 0.8), and perceived behavioral control (α = 0.6) were considered acceptable and good internal consistency. We used logistic regression to measure the association of intention to the smoking behavior change and the association between intention and the determinants. Data were analyzed by Stata Statistical Software Release 12 licensed to the Department of Public Health, Faculty of Medicine, Universitas Gadjah Mada.

## Results

### The Smoking Cessation Program Titled “Smoke-Free KPC 2015”

KPC started executing a massive smoking cessation multi-component program at the beginning of 2014 to achieve a smoke-free worksite in January 2015. More than 1,000 people participated in various activities, including advocacy, health education, training, small group discussion, and a seminar. Health education was applied to all levels, from top management to field workers, using various events. More than 200 managers, supervisors, and superintendents attended the main meeting to launch the multi-component smoking cessation program. KPC also implemented health education for staff's families, mainly for workers' wives, students in schools surrounding KPC, and the community in Sangatta, a small district in East Kalimantan where KPC is located. More than 300 participants consisting of school teachers and principals, mining contractors, government officials, and community representatives attended a 1-day seminar proposed to the local government to make Sangata healthy. The resource persons of the seminar consisted of tobacco control experts from the well-respected Faculty of Medicine, Public Health and Nursing, Universitas Gadjah Mada in Yogyakarta, Indonesia, and the CEO of KPC. The highlight of the sharing session was testimony from one staff of KPC who used to be a smoker and quit smoking due to having suffered from a heart attack.

The Health Safety Environment and Security Division conducted advocacy to the top management to support and reinforce the smoke-free policy. To show the effect of smoking, they also downloaded several short videos and televised them on the bus that takes mining workers to the worksite every day. In addition, twenty ex-smokers were trained in a 2-day training as peer educators to help the employee smokers quit. Based on the discussion with the peer educators 6 months after the training, only a very limited number of smokers consulted with them. However, they tried to talk about quitting smoking with their smoker colleagues in every available chance.

The Smoke-Free KPC 2015 policy has been applied with close monitoring. KPC even applied random screening of bringing cigarettes and matches amongst employees. Supervisors often remind their workers about not smoking and the importance of quitting smoking. Management has also reminded employees about the smoke-free area policy in every meeting.

Big billboards about Smoke-Free KPC 2015 have been placed in several strategic locations in the mining area. Posters were put up in the KPC clinic, dormitory, and canteen. Leaflets were also given to the attendees of health education sessions and made ready to be given to all patients who visited the KPC clinic.

### The Evaluation Survey

A total of 404 workers participated in the evaluation survey conducted in 2016; only 363 could be analyzed (Table 1). The majority of the respondents were male (89.8%), married (87.3%), and lived with family (80.7%). The proportion of smokers was 28% of the KPC workforce.

**Table 1:** Respondents' characteristics (*n* = 363).

**Variables**		***n***	**%**
**Sex**			
Male		326	89.8
Female		37	10.2
Age (mean SD)		36.7	8.3
**Marital status**			
Unmarried and widowed		46	12.67
Married		317	87.33
**Living**			
Family		293	80.7
Alone/dorm		70	19.3
**Smoking status**			
Non-smoker		161	44.4
Quitter		100	27.6
Smoker		102	28.0

*SD, standard deviation*.

Knowledge of smoke-free legislation at the provincial and company level is presented in Table 2. Almost all of the participants attended the company education and socialization campaigns. There was no difference in the knowledge between the type of smokers. Only half of the participants reported that they knew the new smoking laws were enacted by the Governor of East Kalimantan. Higher proportions of workers (more than 90%) said they have been aware that KPC is a 100% smoke-free area.

**Table 2:** Knowledge about smoke-free policy.

			**Non-smoker**	**Quitter**	**Smoker**	**Total**	***p*-value**
Ever had company socialization	Yes	*N*	153	98	96	347	0.396
		%^*^	95.0	98.0	94.1	95.6	
Knowledge of Provincial SFA regulation	Yes	*N*	76	50	50	176	0.901
		%^*^	47.2	50	49.0	47.5	
Knowledge of workplace is SFA by Provincial SFA regulation	Yes	*N*	81	51	52	184	0.992
		%^*^	50.3	51.0	51.0	50.7	
Knowledge if KPC is SFA by company regulation	Yes	*N*	160	97	101	358	0.231
		%^*^	99.4	97.0	99.0	98.65	

*Percentages represent those participants who answered “yes”.

**Respondents'**' views on policy implementation of KPC” SFA Regulation are presented in Table 3. Almost all non-smokers and 89% of quitters perceived that workers should not be allowed to smoke in the workplace, compared to only two-thirds of the smoker (*p* < 0.001). The majority of the non-smokers (93.2%) and quitters (82%) also believe that KPC's SFA regulation should have been enacted earlier. No significant difference was observed in the perceptions that staff members have understood the KPC” SFA regulation (non-smoker 83.2%, quitter 78%, smoker 73.5%; *p* = 0.162) and have implemented the KPC” SFA regulation (non-smoker 54.7%, quitter 50%, smoker 49%; *p* = 0.615). While 96.3% of non-smokers and 95.0 of quitters agreed or strongly agreed that everyone in KPC was supposed to obey the KPC SFA regulation, only 82.4% of the smokers expressed their agreement (*p* < 0.001). Concerning having smoking areas in the workplace, about a third of non-smokers (29.8%) and quitters (36%) agree with the statement compared to 71.6% of smokers (*p* < 0.001). The proportion of smokers who agreed that any place in KPC, including the outdoor area, was supposed to be SFA was less (58.8%) than their counterparts (non-smoker 86%, quitters 77%; *p* < 0.001).

**Table 3:** Respondents'ts' views on policy implementation KPC' SFA Regulation – percentage agreement for non-smokers and smokers, % Agreement (Strongly Agree/Agree), by smoking status (*n* = 363).

		**Non-smoker**	**Quitter**	**Smoker**	***p*-value**
The workers shouldn't be allowed to smoke in the working place.	*n*	155	89	68	<0.001
	%	96.3	89.0	66.7	
KPC' SFA regulation should be enacted earlier	*n*	150	82	69	<0.001
	%	93.2	82.0	67.7	
Staff have understood the KPC' SFA regulation	*n*	134	78	75	0.162
	%	83.2	78.0	73.5	
Staff have implemented the KPC' SFA regulation	*n*	88	50	50	0.615
	%	54.7	50.0	49.0	
Everyone in KPC supposed to obey the KPC' SFA regulation	*n*	155	95	84	<0.001
	%	96.3	95.0	82.4	
Only KPC staff supposed to obey the KPC' SFA regulation	*n*	129	84	83	0.734
	%	80.12	84.0	81.4	
The workplace should provide a smoking area	*n*	48	36	73	<0.001
	%	29.8	36.0	71.6	
Any place in KPC, including the outdoor area, supposed to be SFA	*n*	139	77	60	<0.001
	%	86.3	77.0	58.8	

*p-value from Chi-square or Fisher exact test*.

Approximately 10 months after the KPC smoke-free regulation implementation, 78.4% of smokers reduced their consumption, and 4.9% of all smokers stated that they had already quit smoking (Table 4). While only half of the smokers reported that they still smoke in KPC (54.9%), the majority of them still smoke outside KPC (91.2%), in homes (88.2%), and public (81.4%). In the model using the Theory of Planned Behavior, we found that knowledge (OR 1.3; 95%, CI 0.8–2.1), attitude (OR 1.9; 95%, CI 1.3–2.9), subjective norm (OR 4.3; 95%, CI 2.1–8.9) and perceived behavioral control (OR 2.7; 95%, CI 1.5–4.6) were positively associated with the intention to stop smoking (Table 5). The intention to stop smoking was also positively associated with decreasing smoking behavior or smoking cessation (OR 3.2; 95%, CI 1.1–9.3).

**Table 4:** Place of smoking, smoking cessation related component (among smokers, *n* = 102).

	***n***	**%**
Smoking behavior change		
Ever stop	5	4.90
Less	80	78.43
Stay same	16	15.69
More (increased)	1	0.98
Place of smoking^a^		
KPC	56	54.90
Outside KPC	93	91.18
Home	90	88.24
Public	83	81.37

*^a^Proportion of respondents answering: always, often, sometimes, and seldom to smoke in the given places*.

**Table 5:** The construct of theory of planned behavior in the intention to stop smoking and smoking behavior change (among smokers, *n* = 102).

	**OR**	**95% CI**	***p*-value**
Construct on the intention to stop smoking			
Knowledge	1.3	0.8–2.1	0.206
Attitude	2.3	1.5–3.8	<0.001
Subjective norm	4.3	2.1–8.9	<0.001
Perceived behavioral control	2.7	1.5–4.6	<0.001
Construct on the decrease and stop smoking behavior change			
Intention to stop smoking	3.2	1.1–9.3	0.032

*OR, odds ratio*.

## Discussion

This evaluation was conducted 10 months after strengthening KPC Smoke-Free 2015, the same period between implementation and the evaluation study by Fichtenberg and Glantz (post 10 months) (29). A smoke-free workplace is a cost-effective, public health approach that encourages the important long-term goals of eliminating tobacco use and SHS exposure. Following KPC implementing the smoke-free legislation, which began in 2010, an evaluation in 2014 revealed smoking behaviors were reduced slightly from 49 to 46%. This more recent study revealed that the smoking rates have dropped to 28%. This substantial decrease is significantly higher compared to other studies (mean 3.8%; 95%, CI: 2.8–4.7%) (29). Fichtenberg and Glantz, who reviewed tobacco control policy, reported that 100% of smoke-free policies decreased smoking prevalence by about 3.8% and reduced consumption by three cigarettes every day (29). The combination of prevalence decreasing effect and reducing the number of cigarettes smoked every day resulted in an average decrease of 1.3 cigarettes per day per staff, equivalent to the relative decrease to 29%.

This difference in smoking prevalence in KPC between 2014 and this study might be due to several reasons. Smoking rates decline significantly in a total ban area, while a partial smoking ban has no significant impact (30). Workplaces that implement complete smoke-free regulations produce twice the effect on consumption and prevalence as policies that still allow smoking in some areas (29). KPC implemented a total ban and applied other tobacco control activities, and closely monitored the policy implementation. Random proactive inspections to prevent workers from bringing cigarettes to the working site were done. Close monitoring of smoking behavior and law enforcement on smoke-free regulations are considered effective (31, 32). Some interventions directed toward individuals include individual peer counseling and education for all stakeholders. Multiple approaches directed toward individual smokers increase the likelihood of quitting smoking (22, 33). A series of health education promotions have also been done among stakeholders and a media campaign all over the company areas. Health education via media has been shown to reduce smoking prevalence (34). Sims et al. in 2014 indicated that tobacco control advertisements on television could reduce smoking proportions in England by 13.5% (35). Furthermore, KPC has held training for ex-smoker staff to become peer educators for smoking cessation counseling. A study showed that peer education was appropriate and considered effective (22), supporting the importance of this effort.

The reduction in smoking occurred because the smoke-free legislation increases support for regulating smoking, reduces the social acceptability of smoking, limits opportunities for smoking, and leads to less socially cued smoking (36). In the U.S., smoke-free regulations and ordinances also reduce non-smokers' exposure to SHS and decrease respiratory symptoms related to exposure (37). In addition, these laws result in decreases in smoking prevalence and total cigarettes consumed by smokers while increasing cessation attempts. A study in California found a dose-response relationship that associated higher smoking cessation rates with more comprehensive laws (38). A study in France also reported that smoke-free regulations decreased smoking prevalence (39). From the findings in recent research, current smokers have less productivity which averaged a 4.5% decrease (40). Thus, quitting smoking also benefits the company directly by ensuring productivity.

Effective implementation of smoke-free regulations is still challenging in developing countries, such as Nigeria (41) and Bangladesh (42). Moreover, Indonesia is placed as the fourth most populous smoker country globally and the seventh highest in cigarette production (43), while the tobacco control policy remained in its infancy, particularly before 1990 (44). Although an Indonesian delegation participated in developing the Framework Convention on Tobacco Control (FCTC), Indonesia is the only country in the Asia Pacific region that has not ratified the FCTC. Up to April 2013, FCTC has been signed by 173 countries (43). While Indonesia has not signed the FCTC, in 1999, the government issued Regulation of Indonesia number 81, and it was aimed to regulate smoke-free areas in seven settings, including the workplace. The regulation was renewed in 2003 and expanded in 2009 with Government Regulation number 36. That regulation should be followed with local regulations. In 2013, the Ministry of Health of Indonesia reported that ten provinces out of 33 and 127 districts or cities out of more than 500 had issued local regulations on smoke-free areas, and workplace or smoke-free worksite areas should be included in those local regulations (45).

Awareness of smoke-free legislation remains the key to successful implementation (46, 47). The combination of awareness campaigns, legislation, enforcement, and price policies successfully led Finland to implement smoke-free workplaces making a significant tobacco consumption decline (48). Excellent awareness of smoke-free company regulations should be equally followed by awareness on the provincial level because the laws should be applied to everyone (49). In this study survey, the awareness of KPC staff of the company regulations was good, and nearly 100% knew the regulations. A study in Kyrgyzstan among mining employees reported that only 63% (49% women) were aware of tobacco control laws (50). However, only half of the participants were aware of local government regulations. Awareness differences between the provincial and company level policy might be due to the different scope of promotional dissemination. Reduction in smoking behavior inside the company might be followed by smoking in a restricted area outside the company because employees lack knowledge concerning new regulations by the government concerning smoke-free areas in public places.

The findings on the smoking behavior inside the company should also be directly addressed. KPC needs to consider eliminating the possible areas which the staff use to smoke and place extra smoke-free campaign material there (51). Adherence to the smoke-free company regulations should be monitored because other regulations and violations should be addressed continuously with the counseling process (52).

Behavioral changes that occurred after the intervention in this study were further analyzed based on the TPB theory, and the results showed that the behavioral changes that occurred were related to the intention to quit. Meanwhile, attitude, subjective norm, and perceived control have a relationship with intention. This result is in line with the systematic study conducted by Lareyre et al. (23), which shows that the TPB-based interventions have an impact on intentions, attitudes, subjective norms, and perceived behavioral control by 42–50%. A previous study also reported that behavior change interventions based on theory have more promise than interventions without theory (53).

This study has some limitations since this was a cross-sectional study and did not use a randomized controlled trial or a quasi-experimental design. The sample of previous prevalence studies also included all the workers and staff, while this evaluation used a multi-stratification sample.

The cause and effect of the comprehensive tobacco control program cannot be definitively established. The company implemented the comprehensive intervention and cannot be evaluated separately. This study more focussed on the impact of the overall intervention. In addition, the behavior change has been analyzed using the TPB, although the scale for asking the TPB constructs used limited questions taken from the original questionnaire. Further comprehensive questions are needed to assess the variables related to the TPB. Further study is recommended to assess the effectiveness of the intervention. Moreover, this study was conducted in a mining company in East Kalimantan, which may not represent all companies in Indonesia. Therefore, the generalizability of this study is limited. However, even with these limitations, this study is aimed to contribute toward tobacco control programs in Indonesia, particularly in worksite settings, where there is an obvious need for education and socialization programs promoting smoke-free workplaces.

## Conclusions

This study reveals that comprehensive smoke-free regulation impacts awareness and reduces smokers in the mining industry. The findings support tobacco control activities that remain not strongly implemented in Indonesia. Smoke-free workplaces and other settings should be implemented intensively and widely to strengthen tobacco control policies. Therefore, there is a need to advocate for the central and local governments to apply the smoke-free policy.

## Data Availability Statement

The raw data supporting the conclusions of this article will be made available by the authors upon request.

## Ethics Statement

The studies involving human participants were reviewed and approved by Faculty of Medicine's Medical and Health Research Ethics Committee, Universitas Gadjah Mada. The patients/participants provided their written informed consent to participate in this study.

## Author Contributions

YP was responsible for study design, data collection, statistical analysis, and article writing. PP participated in the study design and data collection. BB contributed to the study design article writing and was responsible for the statistical analysis. All authors contributed to the article and approved the submitted version.

## Funding

This work was supported by research grants from the *Dana Masyarakat* (Citizen Fund) Platform from the Ministry of Higher Education and Research, Indonesia. The study's sponsor had no role in study design, data analysis, data interpretation, or report writing. The opinions expressed in this report are not necessarily those of the funders.

## Conflict of Interest

The authors declare that the research was conducted in the absence of any commercial or financial relationships that could be construed as a potential conflict of interest.

## Publisher's Note

All claims expressed in this article are solely those of the authors and do not necessarily represent those of their affiliated organizations, or those of the publisher, the editors and the reviewers. Any product that may be evaluated in this article, or claim that may be made by its manufacturer, is not guaranteed or endorsed by the publisher.
